# Placental O-GlcNAc-transferase expression and interactions with the glucocorticoid receptor are sex specific and regulated by maternal corticosterone exposure in mice

**DOI:** 10.1038/s41598-017-01666-8

**Published:** 2017-05-17

**Authors:** Marie Pantaleon, Sarah E. Steane, Kathryn McMahon, James S. M. Cuffe, Karen M. Moritz

**Affiliations:** School of Biomedical Sciences, The University of Queensland, St Lucia, 4072 Australia

## Abstract

Maternal stress programs offspring disease in a sexually dimorphic manner with males often more adversely affected. Previous studies of maternal glucocorticoid exposure suggest male vulnerability may derive from placental alterations. The hexosamine signalling pathway and O-linked glycosylation (O-GlcNAcylation) are part of an essential adaptive survival response in healthy cells. The key enzyme involved is O-linked-N-acetylglucosamine transferase (OGT), a gene recently identified as a sex-specific placental biomarker of maternal stress. Using a mouse model of maternal corticosterone (Cort) exposure, we examined components of hexosamine biosynthesis/signalling and O-GlcNAcylation in whole placentae at E14.5. Our results demonstrate sex-specific differences in OGT levels and O-GlcNAcylation during Cort exposure which impacts on key mediators of cell survival, in particular AKT as well as the stress responsive OGT/GR transrepression complex. In male placentae only, Cort exposure increased Akt O-GlcNacylation which correlated with decreased phosphorylation. Female placentae had higher basal OGT and OGT/GR complex compared with male placentae. Cort exposure did not alter these levels in female placentae but increased global O-GlcNacylation. In male placentae Cort increased OGT and OGT/GR complex with no change in global O-GlcNacylation. These findings suggest that sex-specific differences in placental OGT play a key role in the sexually dimorphic responses to stress.

## Introduction

A sub-optimal or stressful environment *in utero* has long been recognised as having the potential to alter adult disease propensity^1^. Male fetuses are often reported as being more adversely affected than female fetuses and often demonstrate more severe disease outcomes than their female counterparts. We have previously shown that excessive maternal glucocorticoid administration results in more significant and profound effects on renal and cardiovascular health in male offspring^2^ that appear to derive from sex-specific placental adaptations in response to the same maternal challenge^3, 4^. These sex-specific placental stress responses include differences in placental architecture as well as molecular changes such as altered expression of the enzyme 11β-hydroxysteroid dehydrogenase type 2, which acts as a barrier ameliorating the impact of glucocorticoids. Additionally, altered expression of vasculogenic and other growth factors such as VEGF and IGF-II also occurs in a sex-specific manner. Differences in how male and female placentae respond to stress suggest that sex-specific adaptations of the placenta may be a critical determinant of sexually dimorphic differences in programming outcomes^4–6^.

This leads us to question which stress responses are differentially regulated in placentae of female fetuses compared to male fetuses that may infer these differential outcomes. Recent studies suggest that the X-linked OGT may be a placental biomarker of maternal stress^7^ and also implicated as a driver of sex disparity in disease^8^. This key nucleoplasmic enzyme catalyses the terminal transfer step in the hexosamine signalling pathway which results in the post-translational modification of numerous cellular regulatory proteins in the nucleus and cytoplasm with a single moiety of N-acetyl glucosamine (N-GlcNAc) through O-linked glycosylation (*O-*GlcNAcylation) of key serine and threonine residues^9^. This is a dynamic modification exhibiting properties that are commonly functionally reciprocal to phosphorylation at these same sites to regulate cellular function in response to nutrient and environmental cues^10^. *O-*GlcNAcylation appears to promote cell/tissue survival by regulating a multitude of biological processes including: the phosphoinositide 3-kinase/Akt pathway, heat shock protein expression, levels of reactive oxygen species, ER stress, protein stability, mitochondrial dynamics, and inflammation^11^. Inhibition of hexosamine biosynthesis, which results in the formation of the donor substrate UDP-GlcNAc for O-GlcNAcylation, eliminates cellular stress responsiveness leading to reduced cell survival in numerous systems^12^ including the early mouse embryo^13, 14^. Whilst deletion of the key enzymes involved in hexosamine signalling is found to be lethal in mammals^15–18^, targeted placental deletion of OGT results in profound disruption of early hypothalamic gene expression and elevated hypothalamic-pituitary-adrenal axis responsivity that closely mirrors the phenotype of a mouse model of prenatal stress^19^.

Given the essential role that hexosamine signalling and O-GlcNAc cycling play in regulating the cellular stress and survival response^20^, we sought to examine whether key elements of this signalling system or some of its known targets were implicated in placental adaptations in our model of maternal corticosterone (Cort) exposure in the mouse. We have previously demonstrated that this Cort exposure increases maternal plasma Cort concentrations by approximately threefold compared to Untr levels^4^. We have recently demonstrated a similar magnitude of change in Cort concentrations in rats that were subjected to acute restraint stress^21^ and previous studies have demonstrated a similar relative increase in women with a stressful labour^22^. Using this physiologically relevant model of Cort exposure, our results lead us to conclude that changes in OGT and hexosamine signalling in the placenta in response to maternal stress is likely to be a key mechanism through which sex-specific fetal programming occurs.

## Results

### Maternal physiology, gene and protein expression

**Figure 1:**
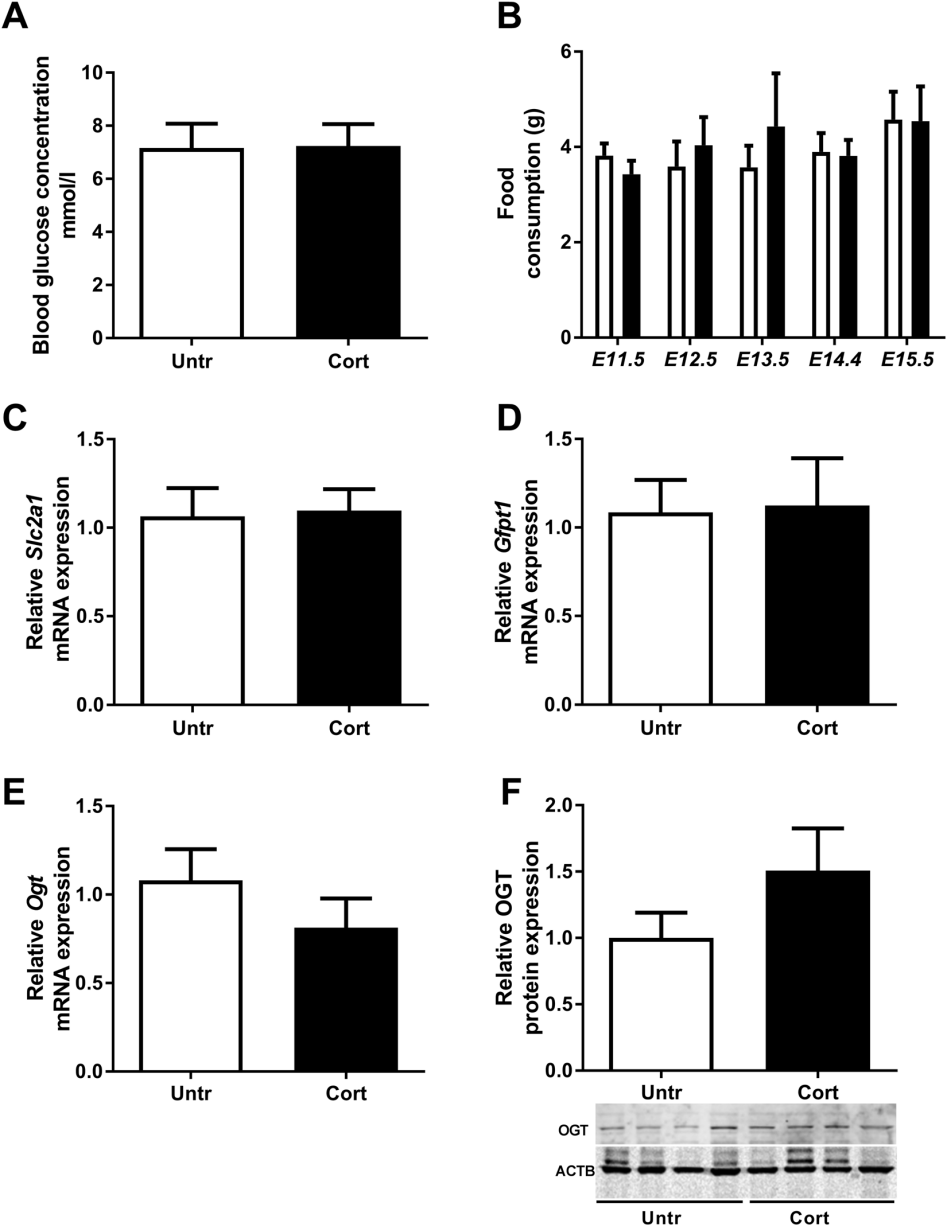
The effects of corticosterone exposure (Cort-black bars) on (**A**) maternal blood glucose concentrations, (**B**) maternal food consumption, (**C**) hepatic mRNA expression of solute carrier family 2, facilitated glucose transporter member 1 (*Slc2a1 or* Glut1), (**D**) Glutamine-fructose-6-phosphate-transaminase 1 (*Gfpt1*) and (**E**) O-linked β-N-Acetylglucosamine (OGT) compared to untreated (Untr-white bars) controls. Protein expression of hepatic OGT was also investigated in Untr or Cort exposed dams (**F**). Data represented as mean + SEM. Maternal blood glucose concentrations and hepatic gene expression results were analysed by two-tailed *t-*tests (n = 6–7). Maternal food consumption was measured daily from E11.5 until E15.5 and data analysed using a one way ANOVA. Protein expression was examined by western blot with β-Actin (ACTB) as a loading control, normalised to untreated control and analysed using a two-tailed *t-*test.

In order to assess if maternal Cort exposure affected maternal physiology, a range of maternal parameters were investigated. Maternal Cort exposure did not affect blood glucose concentrations (Fig. 1A) or food intake (Fig. 1B). In addition, mRNA levels of the glucose sensitive *Slc2a1* (glucose transporter 1 – GLUT1, Fig. 1C), *Pck1* (phosphoenolpyruvate carboxykinase- data not shown), and *Slc2a2* (glucose transporter 2 – GLUT2, data not shown) were not altered significantly by Cort exposure. Gene expression of the rate-limiting enzyme in hexosamine biosynthesis *Gfpt1* (Fig. 1D) and *Ogt* (Fig. 1E) were also not significantly altered in the livers of dams exposed to Cort (n = 6–7). Although OGT protein levels appeared to be slightly elevated in livers of Cort treated dams this was not significantly different (n = 5 per group Fig. 1F).

### Stress responsiveness in the fetal kidney and liver

**Figure 2:**
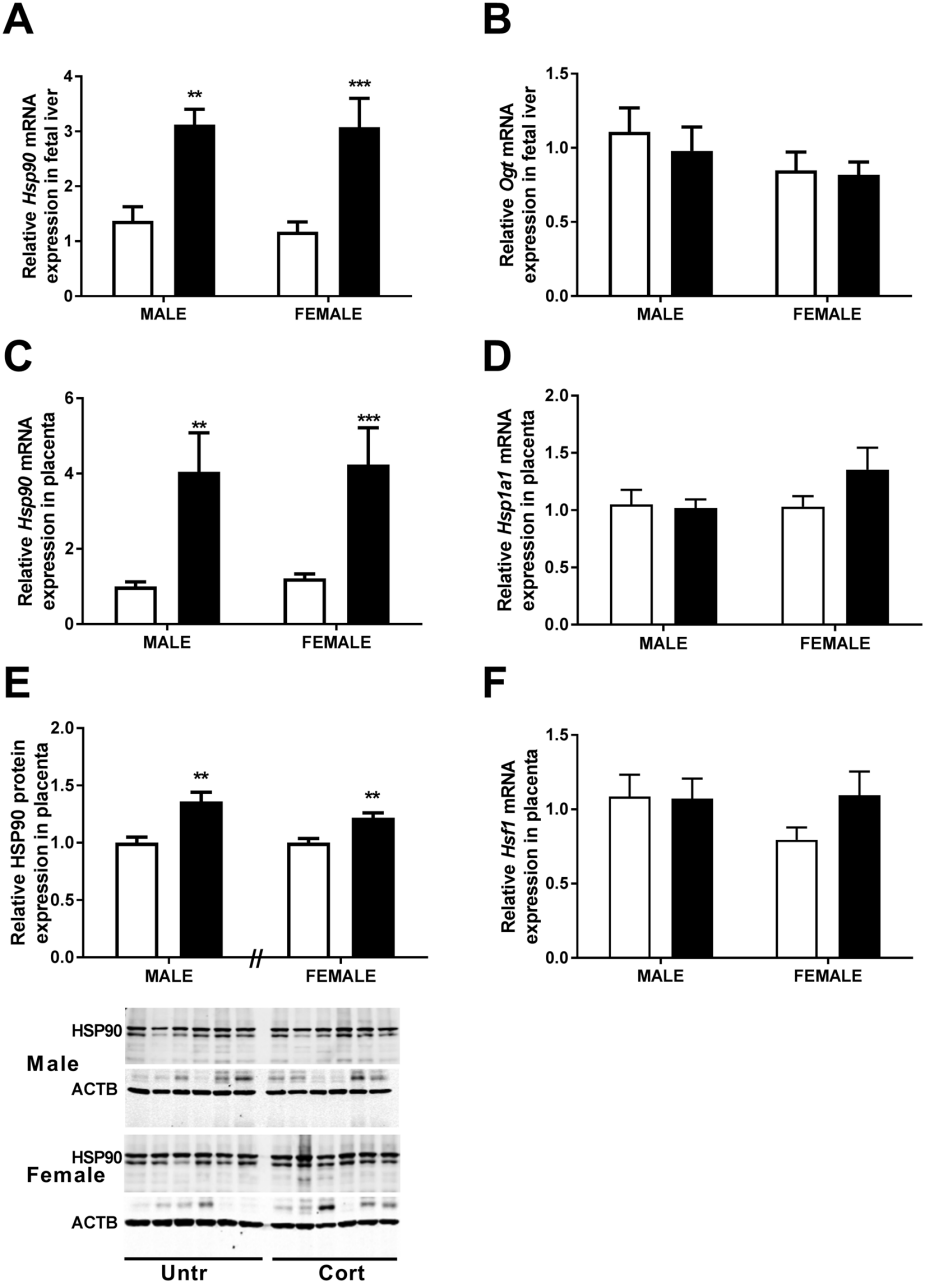
The effects of corticosterone exposure (black bars) on (**A**) relative mRNA expression of Heat Shock Protein 90 (*Hsp90*) and (**B**) O-linked β-N-Acetylglucosamine (*Ogt*) in liver tissue along with (**C**) *Hsp90*, (**D**) Heat shock protein 70 (*Hsp1a1*) and (**F**) Heat Shock Factor 1 (*Hsf1*) in placental tissue of male and female C57/BL6 fetuses at E14.5 compared with untreated controls (white bars). Relative HSP90 protein concentrations were also assessed (**E**) in both male and female placentae at E14.5 (n = 6 per treatment). Data represented as mean + SEM. **P < 0.01, ***P < 0.001 for post hoc analysis or *t-*test results. Real-time quantitative PCR used to determine gene expression, and normalised to β-Actin and 18S RNA levels. RNA data were analysed using a two-way ANOVA comparing the factors treatment and sex with Bonferroni’s *post hoc* testing presented on graph (n = 9–11). Protein expression was examined by western immunoblot with β-Actin (ACTB) as loading control, normalised to the mean of the untreated control of the same sex (Top band analysed for HSP90). Protein expression was analysed using a two-tailed *t-*test with untreated controls of the same sex (n = 6).

To demonstrate that maternal glucocorticoid exposure induced cellular stress in fetal tissues, mRNA expression of a number of stress related genes (*Hsp90*, *Ogt* and *Hsf-1*) was investigated in the fetal liver and kidney. We have previously reported that maternal Cort exposure induced changes in the gene expression in the fetal kidney and heart^2, 23^ but had not investigated stress responsive genes in these tissues. In the current study, *Hsp90a1* was increased in livers of both male and female fetuses (Ptrt < 0.001; Fig. 2A). Given that HSP90 has been shown to effect the regulation of OGT and O-GlcNAc modification^24^, the impact of corticosterone treatment on fetal liver *Ogt* mRNA levels was also examined but found not to be significantly altered in this tissue (Fig. 2B). Maternal Cort exposure did not affect the mRNA levels of *Hsp90a1* (Male Untr = 1.00 ± 0.16, Male Cort = 1.44 ± 0.16, Female Untr = 1.00 ± 0.18, Female Cort = 1.31 ± 0.18), *Hsf1* (Male Untr = 1.03 ± 0.06, Male Cort = 0.97 ± 0.01, Female Untr = 1.11 ± 0.08, Female Cort = 1.03 ± 0.06) or *Ogt* (Male Untr = 1.00 ± 0.07, Male Cort = 0.92 ± 0.03, Female Untr = 0.82 ± 0.12, Female Cort = 0.95 ± 0.09) in the fetal kidney compared to Untr controls.

### Stress responsiveness in the placenta

Placental exposure to stress was also confirmed as indicated by increased expression of *Hsp90a1* mRNA in placentae of both male (P < 0.01) and female (P < 0.001) fetuses of dams exposed to Cort (Ptrt < 0.0001, Fig. 2C). This was also reflected in elevated HSP90 protein levels in placentae from both male and female fetuses exposed to corticosterone as assessed by western immunoblotting (P < 0.01, Fig. 2E). Placental mRNA expression of *Hsp1a1* (Hsp70) and heat shock factor 1 (*Hsf1)* were unaffected at E14.5 during exposure to corticosterone (Fig. 2D and F). Moreover mRNA levels of the transcription factor *Sp1* were also unaffected by Cort treatment (data not shown). We have previously reported that maternal Cort exposure increases mRNA and protein levels of Hsd11b2 in placentas of female fetuses^4^. In the current study, *Hsd11b1* mRNA levels tended to be increased by maternal Cort exposure but this did not reach statistical significance (Ptrt = 0.09, Psex = 0.08, Male Untr = 1.00 ± 0.24, Male Cort = 1.31 ± 0.37, Female Untr = 1.32 ± 0.13, Female Cort = 1.95 ± 0.20).

### Enzymes of the Hexosamine Signalling Pathway in placenta

**Figure 3:**
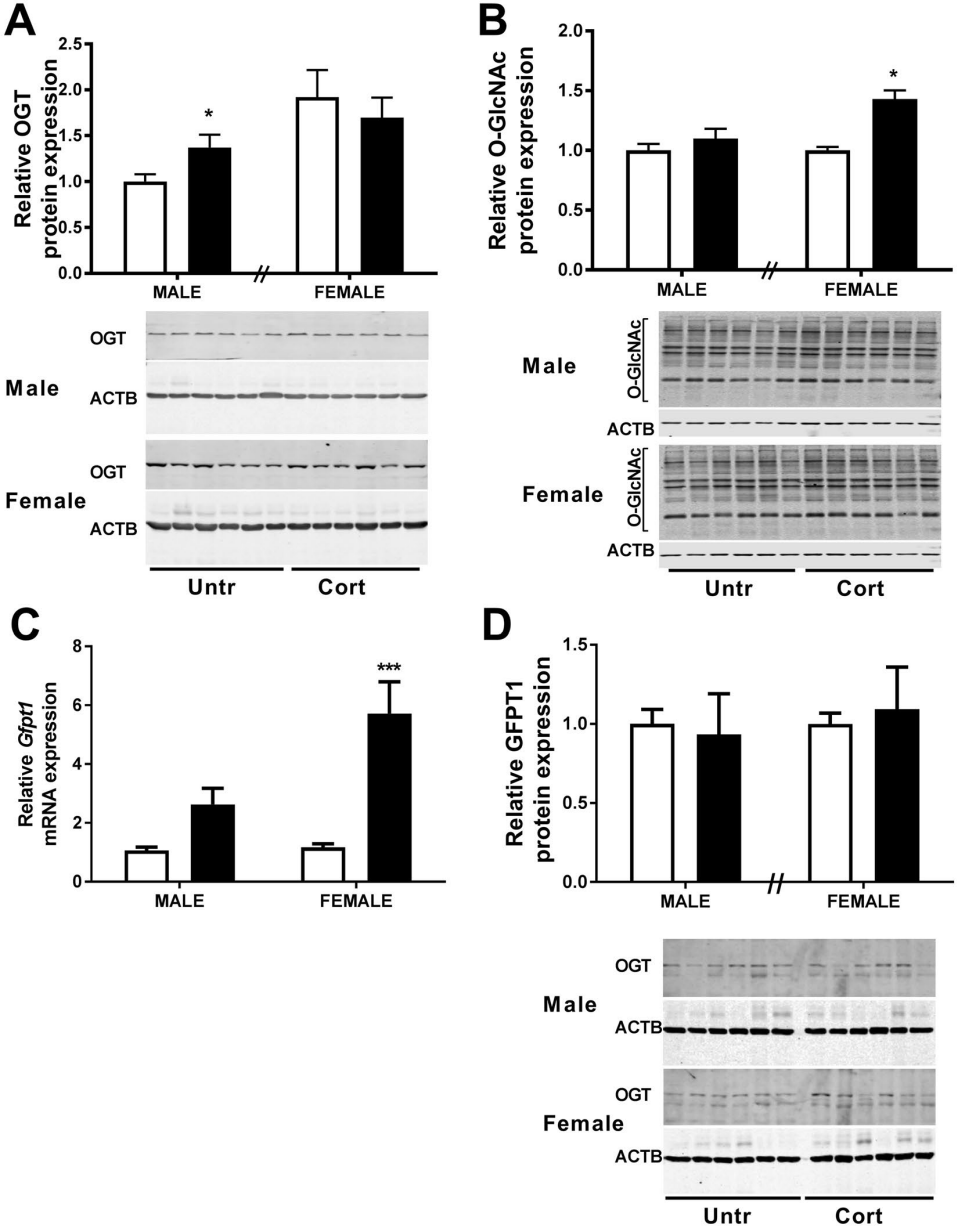
The effects of corticosterone exposure (black bars) on (**A**) O-linked β-N-acetylglucosamine transferase (OGT) relative protein expression and (**B**) levels of O-linked N-acetylglucosamine (O-GlcNAc) modification on cellular proteins in whole placentae of both male and female C57/BL6 fetuses at E14.5 compared with untreated controls (white bars). (**C**) shows glutamine-fructose-6-phosphate-transaminase 1 (GFPT1) relative mRNA and (**D**) GFPT1 protein expression in the same tissues. Data represents mean +SEM, *P < 0.05, ****P < 0.0001 for post hoc analysis or *t-*test results. Real-time quantitative PCR used to determine gene expression, and normalised to the average of two housekeeping genes. RNA data were analysed using a two-way ANOVA comparing the factors treatment and sex with Bonferroni’s *post hoc* testing presented on graph (n = 9–11). Protein expression was examined by western immunoblot with β-Actin (ACTB) as loading control, normalised to untreated control of the same sex. Protein expression was analysed using a two-tailed *t-*test with untreated controls of the same sex (n = 6).

Given that placentas of male and female fetuses had similarly elevated expression of cellular stress markers, we measured expression of the stress regulated protein OGT in placentas of male and female fetuses exposed to Cort. Protein levels of OGT were significantly increased in the placentae of male (P < 0.05; Fig. 3A) but not female fetuses in response to Cort exposure. Western immunoblotting of male and female control placentae on the same gel demonstrated that levels of OGT in female placentae were approximately double those in male placentae (P < 0.05, Fig. 3A, Untr = 1.00 ± 0.30, Cort = 1.92 ± 0.27). Despite this higher level of OGT expression, levels of O-GlcNAcylation, as assessed following western immunoblotting using the RL2 antiserum, were similar in placentae of male and female fetuses from untreated control mothers (Fig. 3B). Surprisingly, global O-GlcNAcylation was not affected by Cort exposure in male placentae but was increased in female placentae by Cort exposure (P < 0.05, Fig. 3B). Given that there were no changes in OGT mRNA levels in either maternal (Fig. 1) or fetal (Fig. 2) livers or kidneys, this suggests that sex-specific adaptive changes in response to stress are specific to the placenta. Although maternal corticosterone exposure increased placental mRNA levels of *Gfpt1* (Ptrt < 0.0001, Fig. 3C), overall, placentae from Cort exposed male fetuses were found to have lower *Gfpt1* mRNA expression than those of Cort exposed females (Psex < 0.05). There was also a significant interaction between sex and treatment and post-hoc analysis demonstrated a significant increase in *Gfpt1* mRNA levels only in female placentae (P < 0.0001). Despite these changes in mRNA levels, GFPT1 protein levels were unaffected by maternal exposure to corticosterone in both male (P = 0.81) and female (P = 0.74) placentae (Fig. 3D).

### Downstream Targets of the Hexosamine Signalling Pathway and O-GlcNAcylation

**Figure 4:**
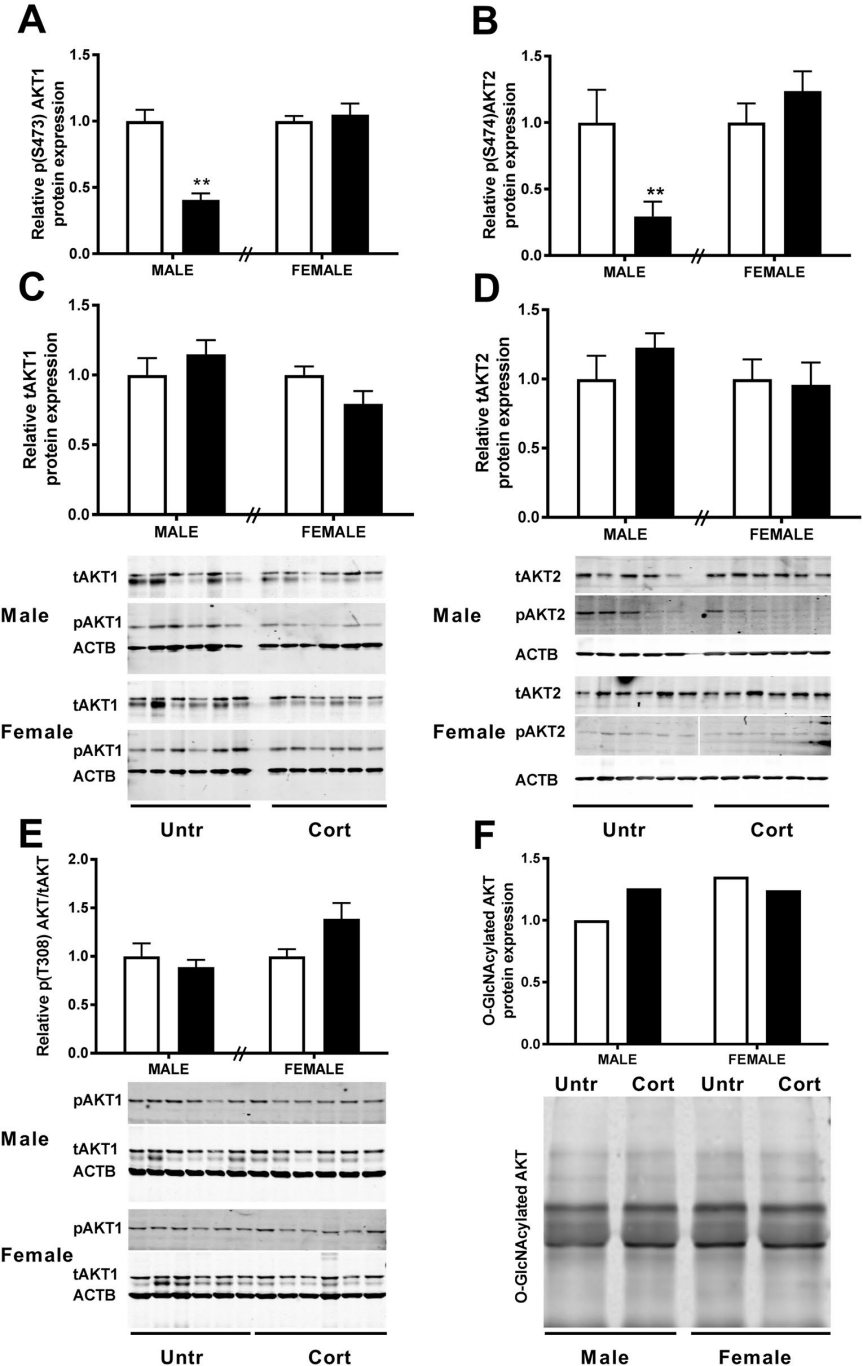
The effects of corticosterone exposure (black bars) on (**A**) serine 473 phospho-AKT1, (**B**) serine 474 phospho-AKT2 along with (**C**) total AKT1 and (**D**) total AKT2 protein in whole placentae of male and female C57/BL6 mouse fetuses at E14.5 compared with untreated controls (white bars). (**E**) Phosphorylation of AKT1 on threonine 308 relative to total AKT1 was also examined. Pooled male and female E14.5 placental tissue (n = 6) was further immunoprecipitated with RL2 antiserum to isolate O-GlcNAcylated proteins and immunoprobed with pan Akt antiserum following SDS-PAGE (**F**). Data represents mean + SEM, **P < 0.01. Protein expression examined by western blot with β-Actin (ACTB) as loading control, normalised to untreated control of the same sex (Top band analysed for tAKT and both bands analysed for O-GlcNAcylated AKT). Analysed using a two-tailed *t-*test with untreated controls of the same sex (n = 6).

Phosphorylation status of both Akt1 and Akt2 on serine 473 and 474 respectively were significantly reduced in male placentae (P < 0.01; Fig. 4A and P < 0.01; Fig. 4B) in response to Cort treatment but maintained at levels similar to control in female placentae. Maternal corticosterone exposure had no effect on total levels of AKT1 and AKT2 protein in the placentae of either sex (Fig. 4C and D). Given that AKT1 is the predominant isoform expressed by the placenta^25^ and is also implicated in placental angiogenesis, we also examined T308 phosphorylation for this isoform. In contrast to S473, this appeared to be unaffected in male placentae in response to stress (P = 0.486; Fig. 4E). In placentae of female fetuses, there was a trend for increased T308 phosphorylation in response to corticosterone treatment however this was not statistically significant (P = 0.0554; Fig. 4E). Experiments using a pan AKT antiserum following immunoprecipitation of O-GlcNAc modified proteins in pooled male placental tissue and pooled female placental tissue at E14.5 demonstrated that levels of O-GlcNAc modified AKT were elevated by approximately 25–30% in male but not female placentae in response to stress (Fig. 4F) reflecting the reciprocal relationship between phosphorylation and O-GlcNAcylation in the regulation of AKT.

### OGT and glucocorticoid receptor interaction and potential transrepression effects

**Figure 5:**
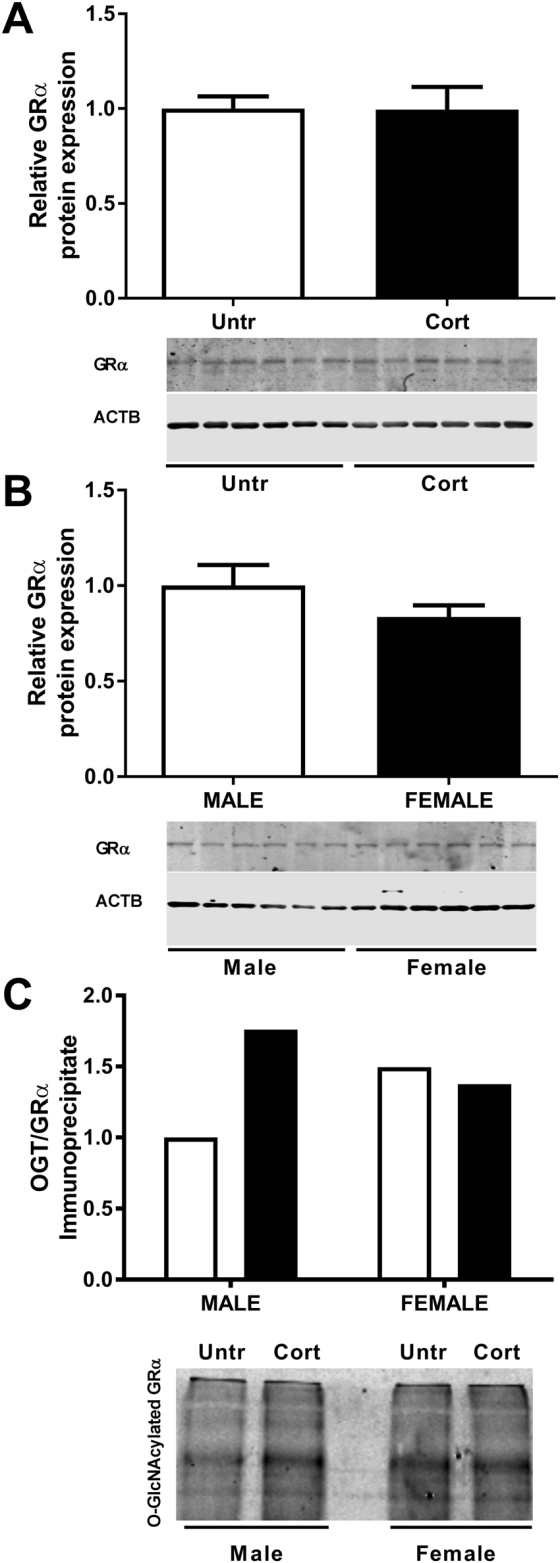
Corticosterone exposure (Cort-black bars) had no effect on (**A**) glucocorticoid receptor alpha (GRα) protein in whole placentae of male C57/BL6 mouse fetuses at E14.5 compared with untreated controls (Untr-white bars) nor was it different between sexes; (**B**) GRα protein in whole placentae of untreated male (white bars) and female (black bars) C57/BL6 mouse fetuses at E14.5. Male and female E14.5 placental tissue (n = 6 pooled for each sex and treatment) were immunoprecipitated to isolate OGT and immunoprobed with a GRα antibody (**C**). This demonstrated an approximate 70% increase in the level of OGT associated glucocorticoid receptor in response to Cort exposure in male fetuses with no apparent difference in the level of OGT immunoprecipitated GRα in female placentae between treatment groups. The level GRα that immunoprecipitated with OGT was approximately 30% greater in placentae of control females than in males.

Using an antibody raised against the glucocorticoid receptor alpha (GRα) isoform of the *Nr3c1* gene we found no difference in the protein level of GRα in response to corticosterone treatment in males (Fig. 5A) nor between the sexes (Fig. 5B). As well as its effects on protein activity via O-GlcNAcylation, OGT has been shown to interact with a number of nuclear factors including the glucocorticoid receptor^26^ to regulate gene expression in response to stress and other environmental cues in cultured cells. We determined that the glucocorticoid receptor does indeed immunoprecipitate with OGT in murine placental tissue (Fig. 5C). Moreover we find that the level of interaction between OGT and the glucocorticoid receptor is approximately 30% greater in females compared to males. Furthermore, Cort administration increased the amount of OGT that immunoprecipitated with the GR by approximately 70% in the placentae of male fetuses. The level of interaction between OGT and the glucocorticoid receptor in females on the other hand appears not to be altered in response to stress.

## Discussion

It is well established that excessive maternal stress as well as synthetic glucocorticoid administration during pregnancy are associated with altered growth and organ development and adverse postnatal outcomes in offspring (for review see ref. 27). Using the same model as utilised in the current study, we have previously demonstrated that maternal Cort exposure induced sexually dimorphic placental adaptations at 48 hours of exposure (E14.5)^4^. These adaptations were characterised by upregulation of the glucocorticoid barrier in females but dysregulated expression of growth and vasculogenic factors such as *Igf2* and *Vegfa* together with structural alterations of the placenta at E17.5 in males only^4^. This was associated with impaired renal^28^, adrenal^29^ and cardiovascular function^2^ only in male offspring. While this suggests that sex differences in the level of fetal glucocorticoid exposure may be contributing to the sex specific disease outcomes, it is clear that placental adaptations mediate these sex differences. In the current study, we have identified sex specific placental adaptations in OGT and associated cell signalling pathways which may provide the female fetus with a greater level of protective capacity to maternal stress and long term disease outcomes^30^.

The Cort exposure in this model increased maternal plasma Cort levels by two-three fold and caused significant negative feedback on maternal adrenal steroidogenesis^4^. We have similarly demonstrated that this increase in maternal Cort levels induced sex specific alterations in glucocorticoid regulated gene and protein expression in the placenta^4^. The current study aimed to determine if this increase in maternal glucocorticoid levels, induced dysregulation of other cellular stress response pathways, which may mediate sex specific outcomes. Firstly, the current study demonstrated that maternal Cort exposure had no effect on maternal food intake or plasma glucose concentrations and did not affect hepatic Gfpt1 or OGT levels. This allowed us to eliminate changes to maternal blood glucose concentrations, hepatic hexosamine signalling or OGT levels as factors contributing to placental and fetal outcomes. We then investigated the effects of maternal Cort exposure on fetal liver and kidney mRNA levels of HSP90 and OGT. The current study demonstrated that *Hsp90aa1* was increased in the fetal liver but not the kidney possibly indicating different protective mechanises (such as HSD11b2 expression) within these two fetal tissues to regulate cellular stress responses. Despite this tissue specific stress response to maternal corticosterone exposure, the fetal response was not affected by fetal sex. Similarly, Ogt levels in both the fetal liver and kidney were not affected by maternal treatment or fetal sex highlighting that sex differences in OGT expression may be specific to the placenta and that sex specific programmed disease outcomes in offspring related to these organs^28^ may occur indirectly as a response to altered placental function.

We then investigated if placentas from male and female fetuses experienced similar levels of cellar stress by examining expression of members of the heat shock protein (HSP) family. Activation of the cellular stress response is usually accompanied by a robust upregulation of heat shock proteins, including HSF-1 and HSP70 (Hsp1a1), which drives increased expression and activity of HSP90^31, 32^. The current study demonstrated that maternal Cort exposure did not alter placental Hsf1 or Hsp70 expression but caused uniform increases in HSP90α both at the gene and protein level in placentae of both sexes. HSP90 is a constitutively expressed protective molecular chaperone involved in suppressing protein aggregation of mis-folded proteins that occurs in response to a range of cellular stresses. In addition, HSP90 can regulate cellular responses to glucocorticoids themselves by facilitating glucocorticoid receptor nuclear translocation and function^33, 34^. This suggests that the sex specific placental adaptations and fetal outcomes are regulated not by the level of cellular stress that the placenta is exposed to but by how the placenta responds to this stress. Indeed, we have previously demonstrated that in response to maternal Cort exposure, mRNA and protein concentrations of the glucocorticoid metabolising enzyme Hsd11b2 are increased to a greater extent in females compared to males^4^. *Hsd11b1* on the other hand, is known to convert inactive glucocorticoids to active glucocorticoids. The current study demonstrates that unlike *Hsd11b2*, placental *Hsd11b1* mRNA levels were not affected by maternal Cort exposure.

A major finding of the current study is that maternal Cort exposure induces sexually dimorphic changes in placental OGT with levels increasing only in the placenta of males. This may be due in part to differences in basal levels of OGT. Bi-allelic expression of *Ogt* in female embryonic stem cells^35^ in accordance with higher resting levels of the gene and protein found in female human placentae^7^, suggests that the *Ogt* gene may escape X-inactivation in the placenta. Consistent with this suggestion we find basal OGT protein levels in the female placenta are twice that of the male. Indeed, the Cort exposure elevated OGT protein levels in the male placenta to similar levels to those observed in the females under basal conditions. The higher basal levels observed in female placentae may therefore be explained by this capacity of the X-linked *Ogt* gene to escape X-inactivation^17^ and importantly, may provide the female fetus with a greater protective capacity, given the rapid ability to respond to stress via this pathway is associated with adaptive cellular survival^20^. It is therefore likely that differential OGT expression and activity may contribute to different disease susceptibility in male versus female offspring in response to stress. In support of this proposition in both humans and animal models where sex chromosome gene dosage is altered as a result of chromosomal abnormalities associated with Turner’s (45X) or Klinefelter’s (47XXY) syndromes, the data suggest that dysregulation of OGT may be implicated in disease disparity^8^. Interestingly, despite no change in OGT in females, there was an overall increase in O-GlcNAcylation in female placenta in response to the Cort exposure. This suggests additional factors other than OGT may contribute to the sex specific alterations in O-GlcNacylation. As discussed further below, we hypothesize a role for the association of OGT with the glucocorticoid receptor. Levels of cellular O-GlcNAcylation increase in response to multiple forms of stress^36^ but must be tightly regulated for cellular homeostasis to be maintained as both inappropriately high and low levels of O-GlcNAcylation are linked to multiple chronic diseases^37^.

Akt is central to development and is a key target of O-GlcNAcylation^38^. The three isoforms (Akt1/2/3) have two major activation sites: T308, T309 and T305 and S473, S474 and S472 respectively. Activity of AKT, which depends upon the degree of phosphorylation at these sites, impacts on cell survival but also processes such as angiogenesis, as seen in endothelial cells^39^ and other stress models^40^. Disruption of AKT signalling results in structural abnormalities of the placenta and intrauterine growth restriction^41^. Previous work in our Cort exposure model resulted in changes in placental morphology consistent with decreased placental efficiency in male but not female fetuses^4^. This was associated with a significant decrease in protein expression of VEGF in male but not female placentae at E14.5, which is likely to have a significant impact on angiogenesis and therefore placental efficiency^4^. In the rat, significant decreases in VEGF were observed in response to synthetic glucocorticoid exposure and this was linked to decreased AKT^S473^ phosphorylation^42^. Those studies however did not examine differences between male and female placentae or provide mechanistic insight as to how this may be mediated. In the current study, although maternal Cort exposure had no impact on total levels of placental AKT1 and AKT2, there was a significant decrease in phosphorylation of AKT1 (AKT1^S473^) and AKT 2 (AKT2^S474^) in the placentas from male fetuses only. This is important as the O-GlcNAc modification is functionally reciprocal to phosphorylation^43^ and as such O-GlcNAc modification of a protein decreases its activity but increases its stability^44–46^. Studies using rat adipocytes have demonstrated this specifically with AKT2, demonstrating that O-GlcNAcylation of AKT2 reduces its activity^38^. Indeed our co-immunoprecipitation experiments in the current study demonstrated that Cort exposure increased O-GlcNAc modification of total AKT in male but not female placentae, confirming the reciprocal nature of this modification and suggesting that the sex specific Cort induced increase in OGT likely mediates this outcome. Collectively this suggests that the decreased phosphorylation of AKT1 at S473 and AKT2 at S474 in males may be occurring by sex-specific alterations of O-glycosylation and may be contributing to the differential alterations in placental growth morphology^47^.

Interestingly, we saw no effect of corticosterone treatment on phosphorylation of AKT1 at T308 in male placentae in response to stress. Maximal activation of AKT1 is regulated by phosphorylation at both T308 and S473 however studies have suggested distinct functions depending on which residue is phosphorylated. While AKT1 is phosphorylated at S473 by TORC2, DNA-PK and TORC1, phosphorylation at T308 is mediated by PDK1 and PIP3^45^. Overexpression of OGT in HEK cells has similarly been shown to reduce phosphorylation levels of AKT at S473 but not at T308^48^. Furthermore, specific blunting of AKT1 activation at T308 through preventing glycosylation of Keratin 18 resulted in apoptosis^49^. It is therefore likely that the Cort induced changes to placental morphology result from OGT induced glycosylation of AKT1 which in turn prevented AKT1 Phosphorylation at S473 to dysregulate placental growth rather than at T308 which may have more strongly affected apoptosis. In contrast, while placentas of female fetuses have higher OGT levels under basal conditions, OGT levels are not affected by Cort exposure and so the phosphorylation status of AKT1 and 2 are similar between both treatment groups.

Given that we have previously demonstrated that Cort exposure leads to the immediate and prolonged upregulation of *Nr3c1* mRNA expression^4^, it was somewhat surprising that GR protein levels were unaffected by prenatal Cort exposure. Previous studies similarly demonstrated that glucocorticoids can increase *Nr3c1* mRNA but not GR protein in the fetal sheep kidney^50^. Given the complex nature of the *Nr3c1* gene (having multiple splice variants and translational isoforms), this discrepancy warrants further investigation but likely indicates that Cort dysregulates placental GR in an isoform specific manner^51^. While classical signalling through the multiple isoforms of the GR can induce a variety of effects on both positive and negative GREs, additional regulation of glucocorticoid mediated cell signalling can be regulated by GR interactions with other protein. Indeed, many nuclear receptors are known to interact with other proteins and transcription factors such as NF-κB to inhibit their function in a process known as transrepression^51^. Recent *in vitro* studies have demonstrated that ligand bound GR interacts with OGT to potentiate GR transrepression pathways in a dose dependant manner^26^. To the best of our knowledge the current study demonstrates for the first time in an *in vivo* model, that there is a direct physical interaction between OGT and the GR. In contrast to the previously discussed *in vitro* studies, our results in placental tissue indicate that the degree of association between OGT and GR is governed by the availability of OGT. This is evident as female placentae have higher basal OGT levels than males and also demonstrate a 30% higher level of OGT/GR complex than male placentae. In addition, in female placentae exposed to Cort, there is no increase in OGT/GR complex formation compared with control placentae. An increase in GR ligand therefore, does not appear to increase complex formation in the placenta. In male placenta on the other hand, Cort exposure does increase OGT protein levels by approximately 40% which in turn increases the OGT/GR complex by approximately 70%.

Interestingly, despite similar levels of OGT/GR complex in control and Cort exposed placentae of females, there was a significant increase in global protein O-GlcNacylation in response to Cort in placentas of female but not male fetuses. Studies have demonstrated that proteins involved in mRNA processing can be O-GlcNacylated which may regulate transcriptional activity^52^. Indeed, GR regulation of the transcriptional regulator NFκB has been shown to be at least partially mediated by the OGT/GR complex *in vitro* as evidenced by increased OGT induced O-GlcNAcylation and decreased phosphorylation of RNA polymerase II^26^. This may also occur *in vivo* whereby glucocorticoids that bind to the OGT/GR complex induce nuclear translocation and direct OGT activity to specific promoter regions, increasing O-GlcNacylation of transcriptional regulators and thereby suppressing transcription of target genes. The findings from the current study suggest that this O-GlcNacylation induced inhibition of gene transcription may be increased in response to Cort only in placenate of females. In contrast, Cort exposure increases the level of OGT/GR interaction but does not give rise to any increase in global protein O-GlcNacylation, perhaps due to insufficient OGT/GR complex upon initial Cort exposure and the requirement to synthesise additional OGT. As there are no basal differences in placental GR protein levels between sexes or between control and Cort exposed male placentae, there is likely to be GR protein which is not complexed with OGT in male placentae. Thus, Cort may bind to “free” GR (Hsp90 bound) to induce nuclear localisation and transcriptional regulation of genes by binding to glucocorticoid responsive elements or interacting with additional coactivator complexes^53^. This proposition is supported by our previous findings of increased expression of a number of genes, such as *Igf2* and *Vegfa* in male but not female placentae upon exposure to Cort. In addition, the increased synthesis of OGT in male placentae in response to Cort, may account for the increased O-GlcNacylation of AKT which would also likely impact gene expression^48^. Although further studies would be useful to confirm subcellular localisation of the OGT/GR complex and O-GlcNac modification, it is clear that there are sex specific differences in OGT levels both basally and in response to Cort.

In conclusion, the current study provides new insight into the mechanisms which underlie the sexually dimorphic placental responses to maternal stress. Our results confirm that placentae of female fetuses have two-fold OGT protein level compared with male placentae. This gives rise to differential complex formation with GR, global O-GlcNacylayion, AKT activity and gene expression, which negatively impacts male placental growth and morphology. This suggests that the higher basal levels of OGT potentiates the OGT/GR complex in female placentae which in turn affords protection by giving greater capacity to rapidly respond to elevated levels of maternal stress.

## Materials and Methods

### Animal treatment

All experiments were approved by The University of Queensland Anatomical Biosciences ethics committee. All experiments were conducted in accordance with the NHMRC guidelines and the Australian Code for the Care and use of Animals for Scientific Purposes. Briefly, Virgin C57BL/6 mice were time mated over a 3-h period with pregnancy being confirmed by the presence of seminal plugs and recorded as embryonic day 0.5 (E0.5). At E12.5, dams were anaesthetized for implantation of an osmotic pump as previously described^4, 28^. Pumps were filled to capacity with Cort and primed for a 60-h infusion (E12.5–E15) at a rate of 33 μg/kg·h. The control group for this study was an untreated group not exposed to the stress resulting from anesthesia and surgery. Food and water intake were measured daily throughout pregnancy and extra care was taken to minimise all stress in both the Untr and Cort groups. On the 14^th^ day of gestation (E14.5) dams were killed at 10am by cervical dislocation (Controls n = 7; Corticosterone n = 7) and blood glucose concentrations immediately collected. Maternal livers were collected and Placentae, livers and kidneys collected from fetuses for analysis. We have previously reported the Cort concentrations of maternal plasma in these pregnant female mice^4^.

### Fetal sex identification

Fetal tails were removed and incubated in lysis buffer with proteinase K overnight at 55 °C prior to heat inactivation. After DNA purification, this extract was analysed using Real-time PCR with primers designed for *Sry* (sex-determining region Y, Mm00441712_s1) as previously described^4^.

### RNA extraction and real-time PCR

RNA was extracted from maternal livers (n = 6–7 per group), fetal livers (n = 9–11 per sex from 6–7 litters), fetal kidneys (n = 6–7 per sex) and placental quarters containing both junctional and labyrinth zone (n = 9–11 per sex from 6–7 litters) using the RNeasy mini kit (QIAGEN, Doncaster, Victoria, Australia). When more than one male or female was used per litter, all results were averaged such that only one value per sex was used per litter. RNA was treated with deoxyribonuclease 1, and reverse transcribed for qPCR analysis using 25 ng of cDNA per reaction^4^. Maternal liver mRNA levels were measured using Assays-on-Demand primer/probe sets (Life technologies, Foster City, CA) for *Slc2a1* (Solute carrier family 2 (facilitated glucose transporter), member 1 (encodes for Glucose Transporter 1 (GLUT1)), Mm 00441473_m1), *Pck1* (Phosphoenolpyruvate carboxykinase 1, Mm 01247058_m1), *Slc2a2* (Solute carrier family 2 (facilitated glucose transporter), member 2 (encodes for Glucose Transporter 2 (GLUT2)), Mm 00446229_m1), *Gfpt1* (Glutamine fructose-6-phosphate transaminase 1, Mm 01183874_m1) and *Ogt* (O-linked N-acetylglucosamine (GlcNAc) transferase, Mm00507317_m1).

mRNA expression of key stress responsive genes were measured in both the fetal liver and kidney. Within the fetal liver, mRNA levels of *Hsp90aa1* (Heat shock protein 90 alpha (cytosolic), class A member 1, Mm00658568_gH) and *Ogt* were obtained. The gene expression of *Hsp90aa1, Ogt and Hsf1 (Heat shock factor 1, Mm01201402_m1*) were measured in the fetal kidney. Placental mRNA levels were measured for *Hsp90aa1, Hspa1a* (Heat shock protein family A, member 1A, encodes HSP70 1a, Mm01159846_s1), *Hsf1*, *Sp1 (trans-acting transcription factor 1*, *Mm00489039_m1*), *Hsd11b1 (Hydroxysteroid 11 beta dehydrogenase 1*, *Mm00476182*_*m1*), *Ogt and Gfpt1*. Real-time PCR results were analyzed using the delta delta cycle of threshold fluorescence method using the most appropriate endogenous controls for each tissue from either *Rn18s* (*18s*) or *Actb*. All groups were compared to the average of the male untreated animal group. All qPCR experiments were performed in triplicate.

### Protein extraction, SDS-PAGE, Western blotting and Immunoprecipitation

Protein was extracted from maternal and fetal livers as well as placentae (n = 6 of each sex and treatment group) using commercial Cell Lysis buffer (Cell Signalling Technologies, #9803) supplemented with protease inhibitor cocktail (Sigma) but also additionally supplemented with sodium fluoride, sodium vanadate and sodium pyrophosphate and O-(2-acetamido-2-deoxy-D-glucopyranosylidene) amino-N-phenylcarbamate (PUGNAc, Sigma Aldrich #A7229; to a final concentration of 0.04 mM), a nonselective inhibitor of O-GlcNAcase to maximize recovery of O-GlcNAc modified proteins. 20 μg of protein was loaded per well in 12% polyacrylamide gels and subjected to SDS-PAGE. Protein was transferred to Immobilon FL polyvinylidene difluoride membranes for Western blotting. After blocking, membranes were incubated overnight at 4 °C with primary antibodies seen in Table 1. LI-COR Biosciences (Lincoln, NE) IRDye 680 goat anti-rabbit and the IRDye 800CW goat anti-mouse secondary antibodies were then used and the Western blots analyzed using the LI-COR Odyssey infrared imaging system (Millennium Science, Surrey Hills, Victoria, Australia). Levels of specific O-GlcNAc modified proteins and OGT/GR interactions were assessed following immunoprecipitation using commercially available Protein G labelled Dynabeads (Life technologies, product number #10004) and specific antisera as per Table 1 using manufacturer’s instructions. Once optimised, all western blots were performed a minimum of 2 times and average values analysed. Immunoprecipitation experiments used pooled samples and were performed a minimum of three times and average values analysed. Unprocessed images of representative western blots are provided in supplementary information.

**Table 1:** Antibodies used for immunoblotting and precipitation.

Full Name	Abbreviation	Raised in	Company	Product No.	Dilution
Glutamine fructose-6-phosphate transaminase 1	GFPT1	rabbit	Abcam	Ab125069	1:1000
O-linked N-acetylglucosamine (GlcNAc) transferase	OGT	rabbit	Cell Signalling Technology (CST)	5368S	1:1000 WB
					1:25 IP
O-linked N-acetylglucosamine	O-GlcNAc (CTD110.6)	mouse	CST	9875S	1:1000
O-linked N-acetylglucosamine	O-GlcNAc (RL2)	mouse	Abcam	ab2739	1:000 WB
					1:20 IP
Heat Shock Protein 90	HSP90	rabbit	CST	4877S	1:1000
Protein Kinase B 2	AKT2	rabbit	CST	3063P	1:800
Serine474-phosphorylated Protein Kinase B2	Phospho- AKT2(Ser474)	rabbit	CST	8599S	1:800
Protein Kinase B 1	AKT1	mouse	CST	2967	1:1000
Serine473-phosphorylated Protein Kinase B1	Phospho- AKT1(Ser473)	rabbit	CST	4060	1:1000
Threonine308-phosphorylated protein kinase B1	Phospho-Akt (Thr308)	Rabbit	CST	9275	1:1000
Glucocorticoid receptor (D8H2)	GCR	Rabbit (mAb)	CST	3660P	1:000

### Statistical analysis

Results are represented as the mean ± sem. Statistical analyses were performed using GraphPad Prism version 6 for Mac. Gene expression results were analysed by two-way ANOVA with treatment (*P*trt) and sex (*P*sex) as factors and either Bonferroni or Tukey’s *post hoc* multiple comparison tests were carried out as appropriate. For levels of gene expression in maternal liver two-tailed student’s *t* tests were used between untreated control and corticosterone treated animals. Western blots were analysed using Student’s *t* tests between Control and Cort groups of the same sex or between male and female Control groups. For gene expression data, if more than one animal per litter were used in a group, data was analysed using litter averages such that n = 7 for both the Untr and Cort group per sex.

## Electronic supplementary material

Supplementary Information

## References

[1] Barker DJ (1990). The fetal and infant origins of adult disease. BMJ..

[2] O’Sullivan L (2015). Excess prenatal corticosterone exposure results in albuminuria, sex-specific hypotension, and altered heart rate responses to restraint stress in aged adult mice. Am J Physiol Renal Physiol.

[3] Cuffe JS, Dickinson H, Simmons DG, Moritz KM (2011). Sex specific changes in placental growth and MAPK following short term maternal dexamethasone exposure in the mouse. Placenta.

[4] Cuffe JS, O’Sullivan L, Simmons DG, Anderson ST, Moritz KM (2012). Maternal corticosterone exposure in the mouse has sex-specific effects on placental growth and mRNA expression. Endocrinology.

[5] Gardebjer EM, Cuffe JS, Pantaleon M, Wlodek ME, Moritz KM (2014). Periconceptional alcohol consumption causes fetal growth restriction and increases glycogen accumulation in the late gestation rat placenta. Placenta.

[6] Clifton VL (2010). Review: Sex and the human placenta: mediating differential strategies of fetal growth and survival. Placenta.

[7] Howerton CL, Morgan CP, Fischer DB, Bale TL (2013). O-GlcNAc transferase (OGT) as a placental biomarker of maternal stress and reprogramming of CNS gene transcription in development. Proc Natl Acad Sci USA.

[8] Abramowitz LK, Olivier-Van Stichelen S, Hanover JA (2014). Chromosome imbalance as a driver of sex disparity in disease. J Genomics.

[9] Holt GD, Hart GW (1986). The subcellular distribution of terminal N-acetylglucosamine moieties. Localization of a novel protein-saccharide linkage, O-linked GlcNAc. J Biol Chem..

[10] Hart GW, Housley MP, Slawson C (2007). Cycling of O-linked beta-N-acetylglucosamine on nucleocytoplasmic proteins. Nature..

[11] Groves, J. A., Lee, A., Yildirir, G. & Zachara, N. E. Dynamic O-GlcNAcylation and its roles in the cellular stress response and homeostasis. *Cell Stress Chaperones*. **18**, 535–558. doi:510.1007/s12192-12013-10426-y, Epub 12013 Apr 12126 (2013). 10.1007/s12192-013-0426-y PMC3745259 23620203

[12] Zachara, N. E. & Hart, G. W. Cell signaling, the essential role of O-GlcNAc! *Biochim Biophys Acta*. **1761**, 599–617, Epub 2006 May 2006 (2006). 10.1016/j.bbalip.2006.04.007 16781888

[13] Pantaleon M, Scott J, Kaye PL (2008). Nutrient sensing by the early mouse embryo: hexosamine biosynthesis and glucose signaling during preimplantation development. Biol Reprod.

[14] Pantaleon M (2015). The role of hexosamine biosynthesis and signaling in early development. Adv Exp Med Biol.

[15] Boehmelt G (2000). Decreased UDP-GlcNAc levels abrogate proliferation control in EMeg32-deficient cells. EMBO J..

[16] O’Donnell N, Zachara NE, Hart GW, Marth JD (2004). Ogt-dependent X-chromosome-linked protein glycosylation is a requisite modification in somatic cell function and embryo viability. Mol Cell Biol.

[17] Shafi R (2000). The O-GlcNAc transferase gene resides on the X chromosome and is essential for embryonic stem cell viability and mouse ontogeny. Proc Natl Acad Sci USA.

[18] Yang, Y. R. *et al*. O-GlcNAcase is essential for embryonic development and maintenance of genomic stability. *Aging Cell*. **11**, 439–448, doi:410.1111/j.1474-9726.2012.00801.x, Epub 02012 Feb 00828 (2012). 10.1111/j.1474-9726.2012.00801.x 22314054

[19] Howerton CL, Bale TL (2014). Targeted placental deletion of OGT recapitulates the prenatal stress phenotype including hypothalamic mitochondrial dysfunction. Proc Natl Acad Sci USA.

[20] Zachara, N. E. *et al*. Dynamic O-GlcNAc modification of nucleocytoplasmic proteins in response to stress. A survival response of mammalian cells. *J Biol Chem*. **279**, 30133–30142, Epub 32004 May 30111 (2004). 10.1074/jbc.M403773200 15138254

[21] Spiers JG, Chen H-JC, Cuffe JSM, Sernia C, Lavidis NA (2016). Acute restraint stress induces rapid changes in central redox status and protective antioxidant genes in rats. Psychoneuroendocrinology.

[22] Van Cauwenberge JR (1987). Changes in fetal and maternal blood levels of prolactin, cortisol, and cortisone during eutocic and dystocic childbirth. Hormone research.

[23] Cuffe JS, Steane S, Moritz KM, Paravicini TM (2015). Differential mRNA expression and glucocorticoid-mediated regulation of TRPM6 and TRPM7 in the heart and kidney throughout murine pregnancy and development. PLoS One.

[24] Zhang, F., Snead, C. M. & Catravas, J. D. Hsp90 regulates O-linked beta-N-acetylglucosamine transferase: a novel mechanism of modulation of protein O-linked beta-N-acetylglucosamine modification in endothelial cells. *Am J Physiol Cell Physiol*. **302**, C1786–1796. doi:1710.1152/ajpcell.00004.02012. Epub 02012 Apr 00011. (2012). 10.1152/ajpcell.00004.2012 PMC3378080 22496241

[25] Kent, L. N., Ohboshi, S. & Soares, M. J. Akt1 and insulin-like growth factor 2 (Igf2) regulate placentation and fetal/postnatal development. *Int J Dev Biol* **56**, 255–261. doi:210.1387/ijdb.113407lk. (2012). 10.1387/ijdb.113407lk PMC3894249 22562201

[26] Li MD (2012). O-GlcNAc transferase is involved in glucocorticoid receptor-mediated transrepression. J Biol Chem.

[27] Singh RR, Cuffe JS, Moritz KM (2012). Short- and long-term effects of exposure to natural and synthetic glucocorticoids during development. Clin Exp Pharmacol Physiol.

[28] Cuffe JS, Burgess DJ, O’Sullivan L, Singh RR, Moritz KM (2016). Maternal corticosterone exposure in the mouse programs sex-specific renal adaptations in the renin-angiotensin-aldosterone system in 6-month offspring. Physiological reports.

[29] Cuffe JS, Turton EL, Akison LK, Bielefeldt-Ohmann H, Moritz KM (2017). Prenatal corticosterone exposure programs sex-specific adrenal adaptations in mouse offspring. J Endocrinol.

[30] Stark, M. J., Wright, I. M. & Clifton, V. L. Sex-specific alterations in placental 11beta-hydroxysteroid dehydrogenase 2 activity and early postnatal clinical course following antenatal betamethasone. *American journal of physiology. Regulatory, integrative and comparative physiology* **297**, R510–514, doi:00175.2009 (2009). 10.1152/ajpregu.00175.2009 19535674

[31] Hamiel, C. R., Pinto, S., Hau, A. & Wischmeyer, P. E. Glutamine enhances heat shock protein 70 expression via increased hexosamine biosynthetic pathway activity. *Am J Physiol Cell Physiol*. **297**, C1509–1519, doi:1510.1152/ajpcell.00240.02009, Epub 02009 Sep 00223 (2009). 10.1152/ajpcell.00240.2009 PMC2793053 19776393

[32] Tang S (2016). The interactive association between heat shock factor 1 and heat shock proteins in primary myocardial cells subjected to heat stress. Int J Mol Med.

[33] Kirschke, E., Goswami, D., Southworth, D., Griffin, P. R. & Agard, D. A. Glucocorticoid receptor function regulated by coordinated action of the Hsp90 and Hsp70 chaperone cycles. *Cell*. **157**, 1685–1697, doi:1610.1016/j.cell.2014.1604.1038 (2014). 10.1016/j.cell.2014.04.038 PMC4087167 24949977

[34] Pratt, W. B., Morishima, Y., Murphy, M. & Harrell, M. Chaperoning of glucocorticoid receptors. *Handb Exp Pharmacol*, 111–138. (2006). 10.1007/3-540-29717-0_5 16610357

[35] Lin H (2007). Dosage compensation in the mouse balances up-regulation and silencing of X-linked genes. PLoS Biol..

[36] Zachara NE, Hart GW (2004). O-GlcNAc a sensor of cellular state: the role of nucleocytoplasmic glycosylation in modulating cellular function in response to nutrition and stress. Biochim Biophys Acta..

[37] Bond, M. R. & Hanover, J. A. O-GlcNAc cycling: a link between metabolism and chronic disease. *Annu Rev Nutr* **33**, **205**–**29**, doi:10.1146/annurev-nutr-071812-161240, Epub 072013 Apr 071829 (2013). 10.1146/annurev-nutr-071812-161240 PMC10483992 23642195

[38] Park SY, Ryu J, Lee W (2005). O-GlcNAc modification on IRS-1 and Akt2 by PUGNAc inhibits their phosphorylation and induces insulin resistance in rat primary adipocytes. Exp Mol Med..

[39] Luo, B., Soesanto, Y. & McClain, D. A. Protein modification by O-linked GlcNAc reduces angiogenesis by inhibiting Akt activity in endothelial cells. *Arterioscler Thromb Vasc Biol*. **28**, 651–657, doi:610.1161/ATVBAHA.1107.159533, Epub 152008 Jan 159533 (2008). 10.1161/ATVBAHA.107.159533 PMC2734484 18174452

[40] Ozmen, A., Unek, G., Kipmen-Korgun, D. & Korgun, E. T. The expression of Akt and ERK1/2 proteins decreased in dexamethasone-induced intrauterine growth restricted rat placental development. *J Mol Histol*. **42**, 237–249, doi:210.1007/s10735-10011-19328-10734, Epub 12011 Apr 10723 (2011). 10.1007/s10735-011-9328-4 21512721

[41] Yang, Z. Z. *et al*. Protein kinase B alpha/Akt1 regulates placental development and fetal growth. *J Biol Chem*. **278**, 32124–32131, Epub 32003 Jun 32123 (2003). 10.1074/jbc.M302847200 12783884

[42] Ozmen, A. *et al*. Glucocorticoid exposure altered angiogenic factor expression via Akt/mTOR pathway in rat placenta. *Ann Anat*. **198**, 34–40, doi:10.1016/j.aanat.2014.1010.1007, Epub 2014 Nov 1012 (2015). 10.1016/j.aanat.2014.10.007 25479925

[43] Kelly WG, Hart GW (1989). Glycosylation of chromosomal proteins: localization of O-linked N-acetylglucosamine in Drosophila chromatin. Cell..

[44] Sarbassov DD, Guertin DA, Ali SM, Sabatini DM (2005). Phosphorylation and regulation of Akt/PKB by the rictor-mTOR complex. Science..

[45] Hart JR, Vogt PK (2011). Phosphorylation of AKT: a mutational analysis. Oncotarget.

[46] Yang J (2002). Crystal structure of an activated Akt/protein kinase B ternary complex with GSK3-peptide and AMP-PNP. Nat Struct Biol..

[47] Bozulic, L. & Hemmings, B. A. PIKKing on PKB: regulation of PKB activity by phosphorylation. *Curr Opin Cell Biol*. **21**, 256–261, doi:210.1016/j.ceb.2009.1002.1002, Epub 2009 Mar 1019 (2009). 10.1016/j.ceb.2009.02.002 19303758

[48] Shi J (2012). Diverse regulation of AKT and GSK-3beta by O-GlcNAcylation in various types of cells. FEBS Lett.

[49] Ku, N. O., Toivola, D. M., Strnad, P. & Omary, M. B. Cytoskeletal keratin glycosylation protects epithelial tissue from injury. *Nat Cell Biol*. **12**, 876–885, doi:810.1038/ncb2091, Epub 2010 Aug 1022 (2010). 10.1038/ncb2091 PMC3549664 20729838

[50] Hantzis V (2002). Effect of early glucocorticoid treatment on MR and GR in late gestation ovine kidney. Kidney Int.

[51] Oakley RH, Cidlowski JA (2013). The biology of the glucocorticoid receptor: new signaling mechanisms in health and disease. J Allergy Clin Immunol.

[52] Chaiyawat P, Netsirisawan P, Svasti J, Champattanachai V (2014). Aberrant O-GlcNAcylated Proteins: New Perspectives in Breast and Colorectal Cancer. Front Endocrinol (Lausanne).

[53] Adcock IM, Ito K, Barnes PJ (2004). Glucocorticoids: Effects on Gene Transcription. Proc Am Thorac Soc.

