# Supplementary material for: Placental O-GlcNAc-transferase expression and interactions with the glucocorticoid receptor are sex specific and regulated by maternal corticosterone exposure in mice
Source: Sci Rep. 2017 May 17;7:2017. doi: 10.1038/s41598-017-01666-8 (PMC5435684; doi:10.1038/s41598-017-01666-8)

Supplementary Information

Placental O-GlcNAc-transferase expression and interactions with the glucocorticoid receptor are sex specific and regulated by maternal corticosterone exposure in mice.

Marie Pantaleon, Sarah E Steane, Kathryn McMahon James SM Cuffe* and Karen M Moritz*

***Authors contributed equally**

Raw images of Western blots used for figures 1-5

Figure 1F
OGT

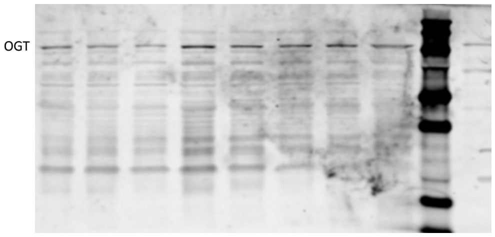

Figure 1F
ACTB

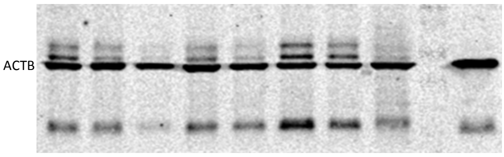

Figure 2F
Males HSP90

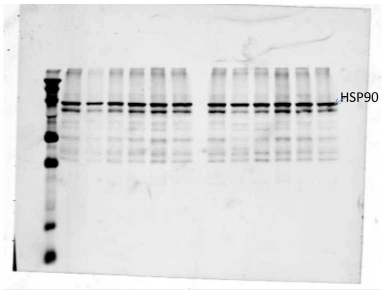

Figure 2F
Males ACTB

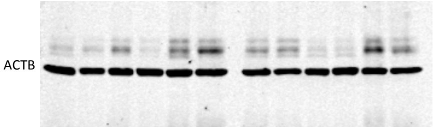

Figure 2F
Females HSP90

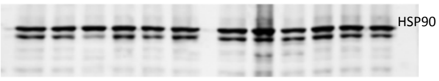

Figure 2F
Females ACTB

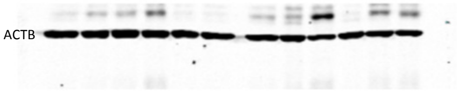

Figure 3A
Males OGT

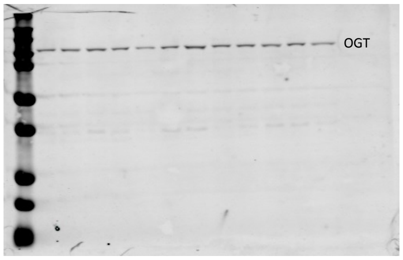

Figure 3A
Males ACTB

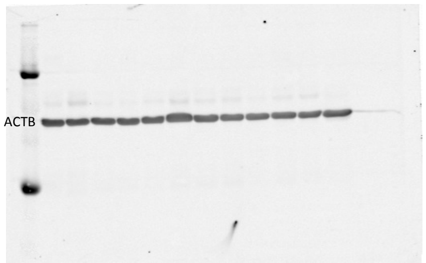

Figure 3A
Females OGT

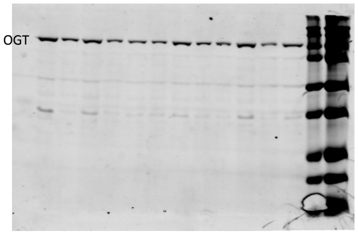

Figure 3A
Females ACTB

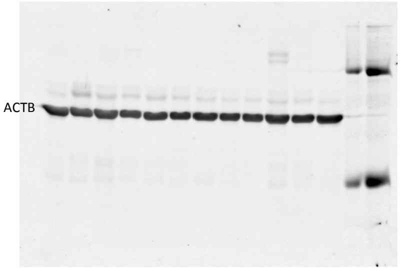

Figure 3B
Males O-GlcNac

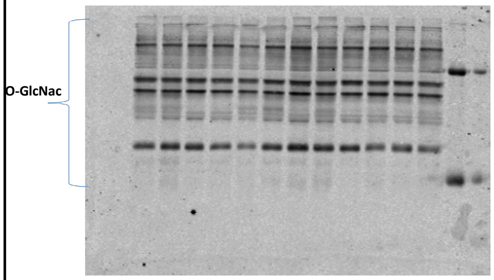

Figure 3B
Males ACTB

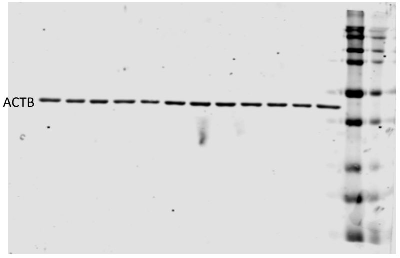

Figure 3B
Females O-GlcNac

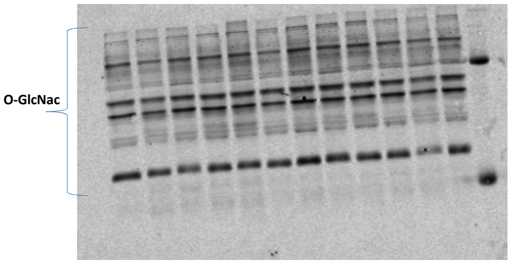

Figure 3B
Females ACTB

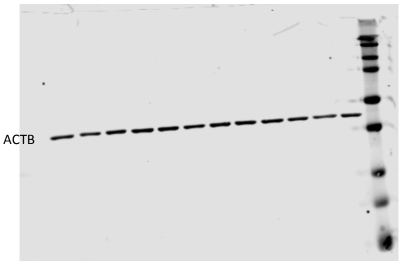

Figure 3D
Male GFPT1

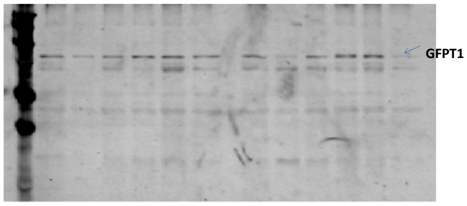

Figure 3D
Male ACTB

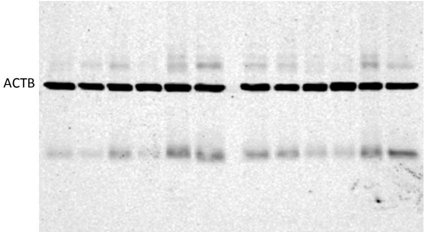

Figure 3D
Female GFPT1

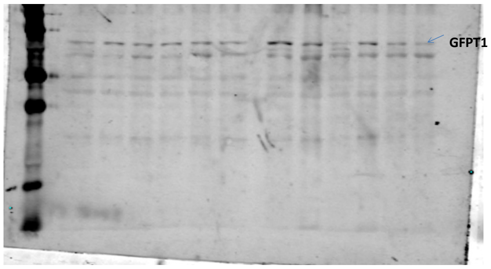

Figure 3D
Female ACTB

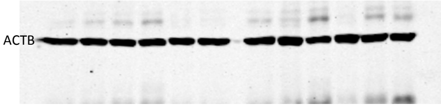

Figure 4A and 4C
Males p(S473) Akt1 and ACTB

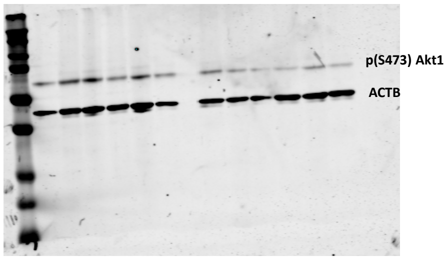

Figure 4A and 4C
Females p(S473) Akt1 and ACTB

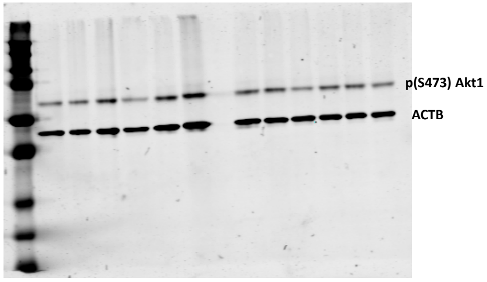

Figure 4C
Males Total Akt1

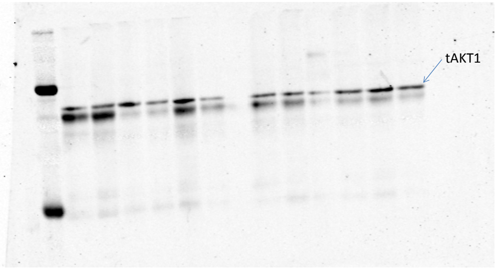

Figure 4C
Females Total Akt1

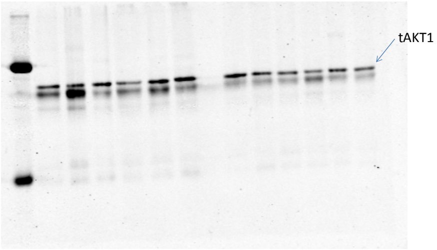

Figure 4B
Males p(S474)Akt2

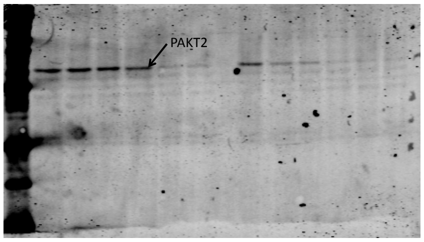

Figure 4B
Females p(S474)Akt2

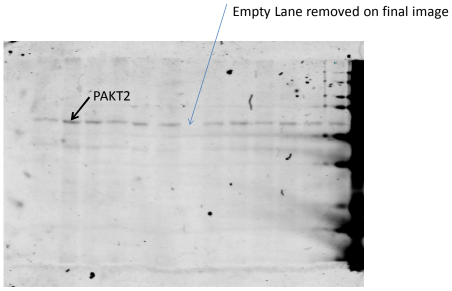

First band excluded to match sample number

Figures 4B and 4D
Males Total AKT2

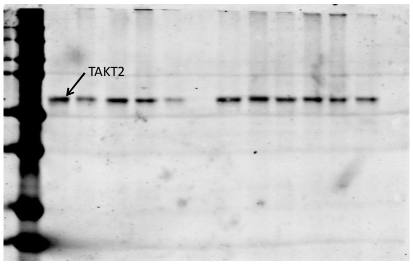

Figures 4B and 4D
Females Total AKT2

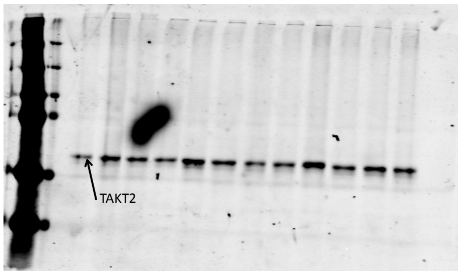

First band excluded to match sample number

Figures 4B and 4D
Males ACTB

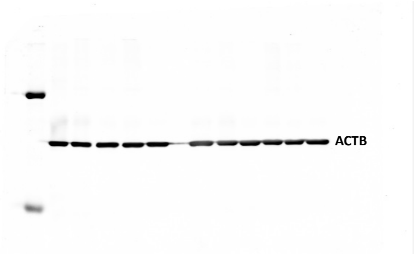

Figures 4B and 4D
Females ACTB

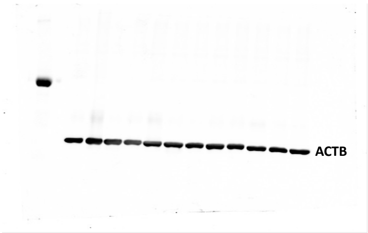

Figure 4E
Males p(T308) AKT

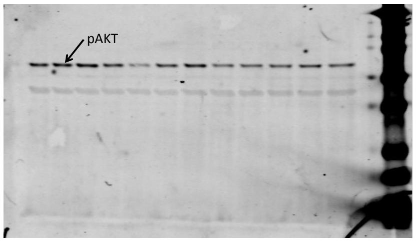

Figure 4E
Males Total AKT and ACTB

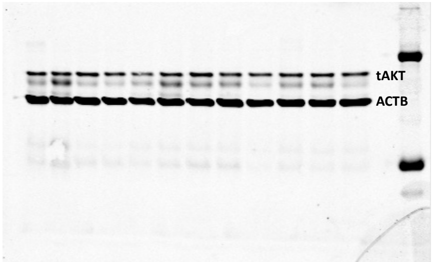

Figure 4E
Females p(T308) AKT

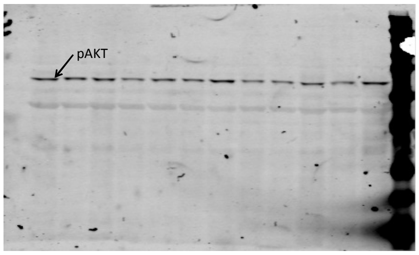

Figure 4E
Males Total AKT and ACTB

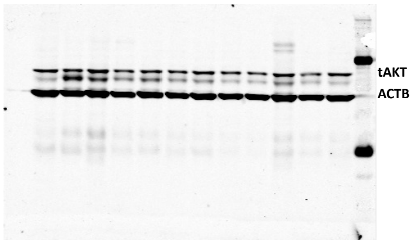

Figure 4F
O-GlcNacylated AKT

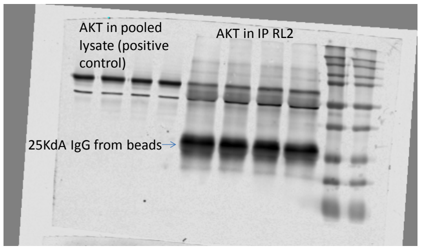

Figure 5A
Males GRα

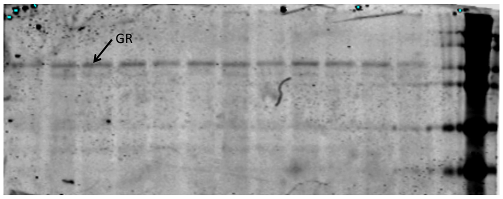

Figure 5A
Males ACTB

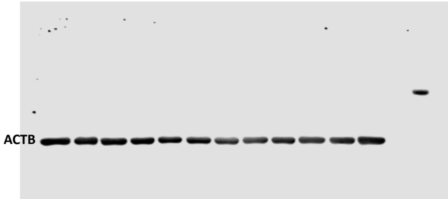

Figure 5B
GRα male and female untreated

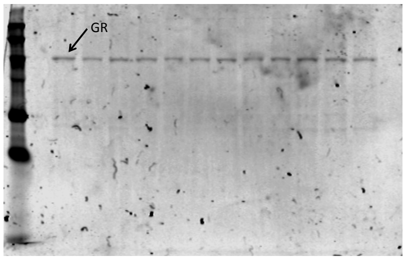

Figure 5B
Male and female untreated ACTB

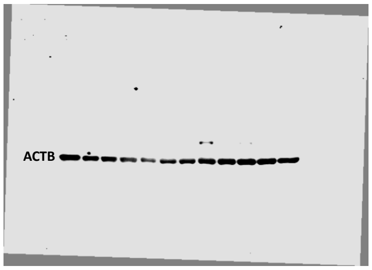

Figure 5C
Males and Females OGT/GRα Immunoprecipitate

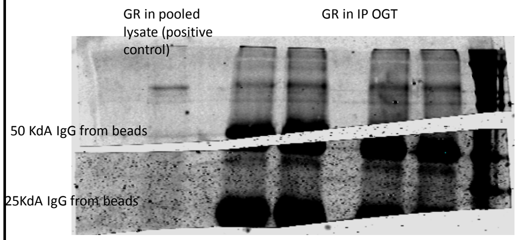

Supplement: Supplementary file 1 — Supplementary Information [file 41598_2017_1666_MOESM1_ESM.pdf]
